# Influence of severe acute respiratory coronavirus virus 2 (SARS-CoV-2) vaccinations on cluster events among patients and staff in a tertiary-care hospital in Germany

**DOI:** 10.1017/ash.2021.234

**Published:** 2022-02-18

**Authors:** Michael Eisenmann, Vera Rauschenberger, Kerstin Knies, Gerhard Schwarzmann, Ulrich Vogel, Manuel Krone

**Affiliations:** 1 Infection Control and Antimicrobial Stewardship Unit, University Hospital Wuerzburg, Wuerzburg, Germany; 2 Institute for Hygiene and Microbiology, University of Wuerzburg, Wuerzburg, Germany; 3 Institute for Virology and Immunobiology, University of Wuerzburg, Wuerzburg, Germany; 4 Quality Control Unit, University Hospital Wuerzburg, Wuerzburg, Germany

## Abstract

In total, 20 severe acute respiratory coronavirus virus 2 (SARS-CoV-2) clusters were analyzed in a tertiary-care hospital from the beginning of the pandemic until July 2021. After the second pandemic wave, the number of clusters decreased with increasing vaccination rates and community infections increased again. These findings should motivate healthcare workers to participate in SARS-CoV-2 vaccination campaigns.

Since the emergence of severe acute respiratory coronavirus virus 2 (SARS-CoV-2) in early 2020, an increasing number of infections have been associated with cluster events.^1^ Due to the high attack rate, SARS-CoV-2 outbreaks could not be avoided entirely, despite the timely implementation of strict hygiene measures and RT-PCR screenings on admission.^2,3^ In this study, we examined the characteristics of nosocomial coronavirus disease 2019 (COVID-19) outbreaks among patients and staff. We sought to determine the causes of the outbreaks, and we analyzed the effect of immunization to outbreak clusters.

## Methods

This study was performed in a tertiary-care hospital in Bavaria, Germany, which provides 1,438 beds for stationary patients and employs ∼7,000 healthcare workers. The superordinate infection control unit of the hospital consists of 14 staff members. The hospital’s vaccination campaign started on December 28, 2020, and ended on June 30, 2021, when 85% of all employees had received their second dose. Staff were vaccinated with the mRNA vaccine BNT162b2 (Comirnaty, Pfizer/BioNTech, New York, NY, and Mainz, Germany), which had proven to be highly effective against symptomatic and asymptomatic infection.^4^ The 7-day incidence per 100,000 population in the state of Bavaria ranged from 2 in June 2020 to 199 in December 2020.^5^

An outbreak was defined as 2 or more epidemiologically linked cases. The cluster ended with the last laboratory-confirmed case. Linear regression analyses were performed using Graph Pad Prism version 9 software (GraphPad Software, San Diego, CA).

## Results

### Cluster events

**Fig. 1.**
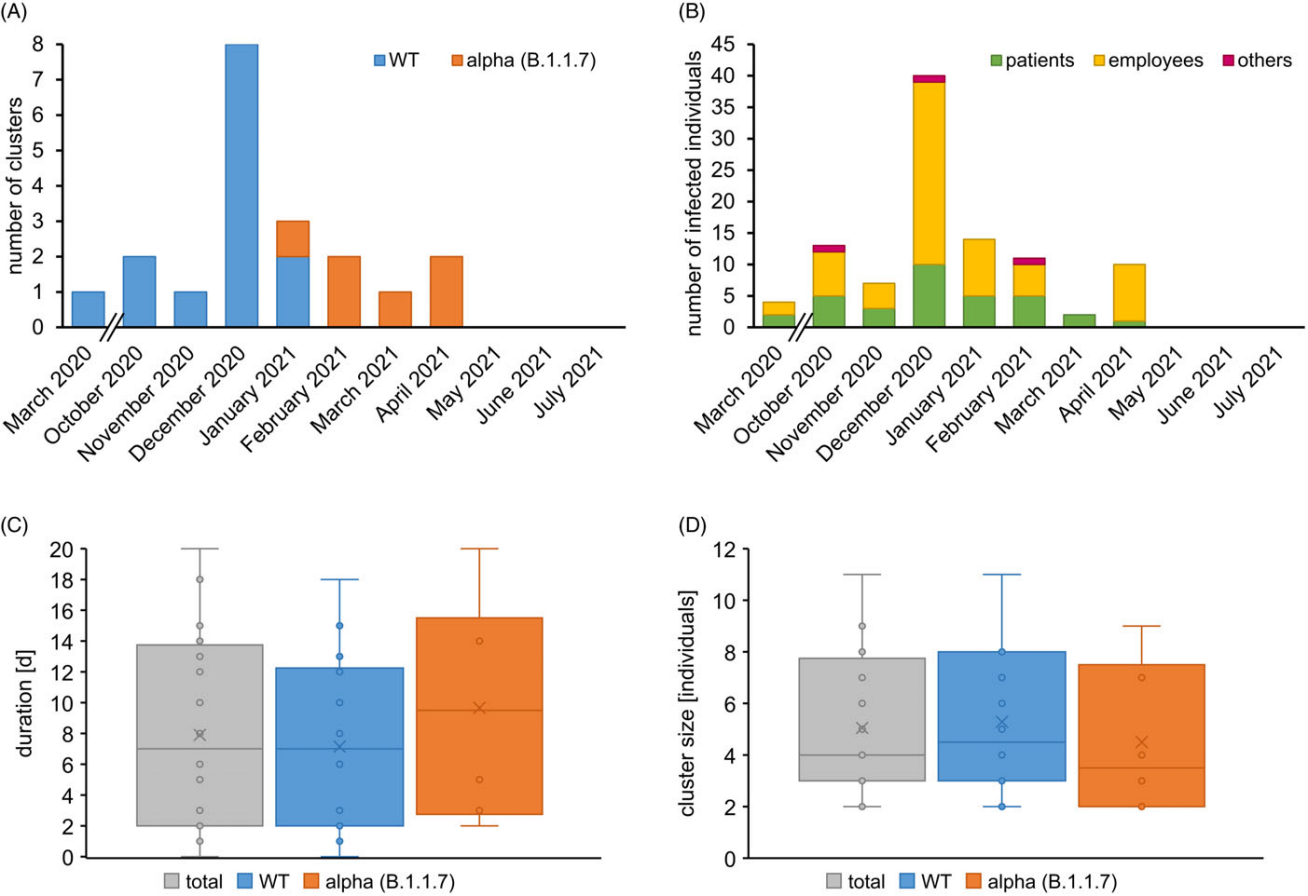
Characteristics of the monthly cluster occurrence. (A) Total number of cluster events per month from March 2020 until July 2021. Clusters were differentiated into wild type (blue) or B.1.1.7 variant (alpha; orange). (B) Total number of individuals in clusters from March 2020 until July 2021. Individuals were divided into patients (green), employees (yellow), and others (purple). (C) Cluster duration for all cluster events (grey) and for clusters with wild-type SARS-CoV-2 (blue) or B.1.1.7. variant (alpha; orange). (D) Same as panel C but describing cluster sizes.

In total, 101 RT-PCR–confirmed SARS-CoV-2 infections in individuals related to outbreaks were documented. Cluster events occurred not only in clinical areas where COVID-19 patients were treated (5 clusters) but also on other wards (12 clusters) as well as in departments outside direct patient care (3 clusters). In December 2020, 8 clusters were detected, the highest number of cluster events at that time (Fig. 1A and 1B). In 11 of 20 outbreaks, index cases were SARS-CoV-2–positive patients (55%), whereas staff members were index cases in 9 of 20 clusters (45%). Among 101 infected persons, 65 (64%) were staff (Fig. 1B), 33 were patients (33%), and 3 (3%) had another role in the hospital (eg, visitor or external technician). Until January 2021, the wild-type variant (including the 614G variant) was the dominant lineage in cluster events and was gradually superseded by the α (alpha) variant (B.1.1.7). Despite the higher attack rate and transmissibility of the α variant,^6^ duration of cluster events did not significantly differ (total: 8 days; wild type: 7 days, α variant: 9 days; *P* = .419) (Fig. 1C). Average cluster sizes were equal (total: 5 cases; wild type: 5 cases; α variant: 5 cases; *P* = .571) (Fig. 1D). No significant trend of cluster size was observed over time (Pearson r = −0.0679; *P* = .776).

### Causes and countermeasures

Although the source of infection could not be determined for some of the events, we identified a multitude of different causes for the 20 outbreaks. These included 4 occurrences of nonadherence to hygiene measures (20%), 2 occurrences of transmission by private contact of staff (10%), and 14 occurrences of transmission among staff and patients in the hospital while presumably meeting hygiene rules (70%). Countermeasures included contact tracing, extended reverse-transcription polymerase chain reaction (RT-PCR) screenings, genotyping of variants, freezing admissions, hygiene training and inspections, fit checks for FFP2 respirators, and a visitor ban. These measures were applied throughout the pandemic, with high compliance during the outbreak, but compliance deficits in hygiene measures were observed outside the outbreaks. Adding rapid antigen testing to the universal PCR screening to detect highly infectious patients as fast as possible was implemented from February to June 2021.^7^ Viral spreading could be interrupted within an average of 8 days.

### Impact of the vaccination campaign

**Fig. 2.**
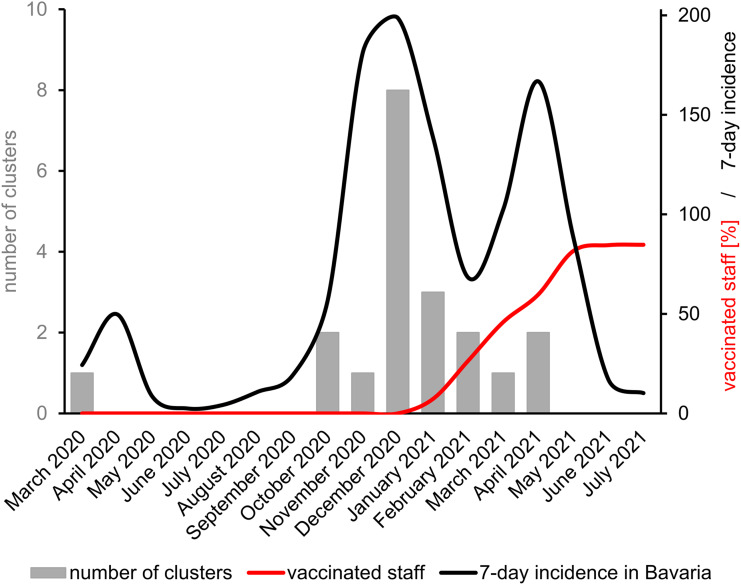
Correlation of cluster events, vaccinated staff, and 7-day incidence in Bavaria. Numbers of clusters are shown as grey bars; percentages of vaccinated staff in the hospital are shown as a red line, and 7-day incidences in Bavaria per 100,000 inhabitants are shown as a black line.

Until April 2021, the number of recorded outbreaks was closely correlated with the 7-day incidence in Bavaria (Fig. 2).^5^ Since March 2021, the total number of cluster events decreased relative to the 7-day incidence and was in line with the constant increase of the number of vaccinated staff and, with considerable delay, vaccinated patients. After May 2021, no further clusters were detected despite the spread of the even more transmissible δ (delta) variant in Germany. Linear regression analysis suggests that the monthly number of clusters was significantly correlated with the 7-day incidence in Bavaria (β = 0.017; 95% CI, 0.010– 0.033; *P* = .001). The number of clusters was negatively correlated with the vaccination status of staff (β = −0.013; 95% CI, −0.036 to 0.009; *P* = .237) as was cluster size (β = −0.020; 95% CI, −0.051 to 0.011; *P* = .193), but these correlations were not significant.

## Discussion

Hygiene measures in combination with centralized outbreak management have proven to be key aspects in pandemic control.^8^ These measures enabled rapid identification of infected individuals and fast disruption of infection chains. Nevertheless, cluster events could not be avoided entirely in our tertiary-care hospital even though universal masking and admission screening of patients became mandatory very early thin the pandemic. Asymptomatic cases and infections that became apparent after admission screening most likely accounted for some of the clusters; other clusters might have originated from insufficient ventilation of rooms. A combination of contact tracing, repeated PCR testing, isolating SARS-CoV-2–positive patients and staff together, and increased awareness of basic hygiene measures are assumed to have been the keys in terminating these outbreaks. Outside the outbreak situation, it is an important to minimize the gap between infection control policies and practice. Before vaccines became available, the number of cluster events reflected the general occurrence of SARS-CoV-2 infection in Bavaria. The rising number of vaccinated staff had a positive, albeit not statistically significant, impact on interrupting this correlation. The number of clusters decreased when the number of community infections in Bavaria increased again. These findings should further motivate healthcare workers to participate in vaccination campaigns to protect themselves, coworkers, and indirectly patients from nosocomial infection.

This study had several limitations: The number of clusters observed was too small and the observation period was too short to assess a statistically significant correlation between number of clusters and vaccination. Information on patients’ vaccination status, which may also have facilitated outbreak control, was not available. This study was observational in design, and the exact impact of basic hygiene measures on the number of infected individuals in clusters could not be accurately quantified because, as in all outbreaks, combinations of multiple measures were applied.

Despite these encouraging findings, a small but increasing number of reported vaccine breakthroughs highlights the importance of combining vaccination campaigns with other infection control measures such as screening, contact tracing, and basic hygiene measures.^9^
