# Influence of severe acute respiratory syndrome coronavirus 2 (SARS-CoV-2) vaccinations on cluster events among patients and staff in a tertiary-care hospital in Germany – CORRIGENDUM

**DOI:** 10.1017/ash.2022.271

**Published:** 2022-08-10

**Authors:** Michael Eisenmann, Vera Rauschenberger, Kerstin Knies, Gerhard Schwarzmann, Ulrich Vogel, Manuel Krone

In the above article^1^, all instances of the following phrase:

“severe acute respiratory coronavirus virus 2”

…have been replaced with the corrected phrasing:

“severe acute respiratory syndrome coronavirus 2”

The publisher apologizes for this error.
